# Endoscopic Ultrasound-Guided Radiofrequency Ablation (EUS-RFA) of the Pancreas in a Porcine Model

**DOI:** 10.1155/2012/431451

**Published:** 2012-09-20

**Authors:** Monica Gaidhane, Ioana Smith, Kristi Ellen, Jeremy Gatesman, Nagy Habib, Patricia Foley, Christopher Moskaluk, Michel Kahaleh

**Affiliations:** ^1^Division of Gastroenterology & Hepatology, Department of Medicine, Weill Cornell Medical College, New York, NY 10065, USA; ^2^School of Medicine, University of Virginia, Charlottesville, VA 22903, USA; ^3^Department of Surgery and Cancer, Imperial College, London SW7 2AZ, UK

## Abstract

*Backgrounds*. Limited effective palliative treatments exist for pancreatic cancer which includes surgery or chemotherapy. Radiofrequency ablation (RFA) uses high frequency alternating current to ablate diseased tissue and has been used to treat various tumors. In this study, we evaluated a prototype probe adjusted to the EUS-needle to perform EUS-RFA to permit coagulative necrosis in the pancreas. *Methods*. Five Yucatan pigs underwent EUS-guided radiofrequency ablation of the head of their pancreas. Using an EUS-needle, RFA was applied with 6 mm and then 10 mm of the probe exposed at specific wattage for preset durations. *Results*. Only one pig showed moderate levels of pancreatitis (20% proximal pancreatitis). The other animals showed much lower areas of tissue damage. In 3 of the 5 pigs, the proximal pancreas showed greater levels of tissue injury than the distal pancreas, consistent with the proximity of the tissue to the procedure site. In 1 pig, both proximal and distal pancreas showed minimal pancreatitis (1%). There was minimal evidence of fat necrosis in intra-pancreatic and/or extra-pancreatic adipose tissue. *Conclusion*. EUS-guided RFA of the pancreatic head with the monopolar probe through a 19-gauge needle was well tolerated in 5 Yucatan pigs and with minimal amount of pancreatitis.

## 1. Introduction

Pancreatic cancer is the fourth leading cause of cancer death in the USA [1]. The 5-year survival rate is only 3% with a median survival of less than 6 months [2]. Conventional treatment approaches, such as surgery, radiation, chemotherapy, or combinations of these, have little impact on the course of this aggressive cancer [3]. Within months of completing chemoradiation, patients frequently have evidence of local tumor progression (biliary or gastric outlet obstruction) or new metastatic disease [3]. Radiofrequency (RF) ablation has been widely used in oncology but not in the pancreas because of its high operative risks [4]. Recent studies have shown the feasibility of monopolar RF ablation in patients with stage III pancreatic cancer in open, percutaneous, or laparoscopic setting [5, 6]. Ablating with endoscopic ultrasound (EUS) guidance allows real-time imaging into the deeply located pancreas [4]. Radiofrequency ablation (RFA) works by emitting energy that uses heat to produce coagulative necrosis in the surrounding tissue [7, 8]. There is a growing interest and need of RFA of the pancreas [4] and it appears that RFA in unresectable pancreatic carcinoma is feasible with acceptable mortality but high morbidity [5, 6, 8–11]. The objective of the study was to report safety and efficacy of EUS-guided transduodenal RF ablation of porcine pancreas using a new well-shaped monopolar probe (Habib EUS RFA, EMcision Ltd., London, UK) that fits better into the EUS needle. The improved needle design should hypothetically permit coagulative necrosis of larger areas of the pancreas, while still minimizing the risk of damage to the intestinal mucosa.

## 2. Procedure 

Five Yucatan pigs (30–35 kgs) were acclimated in the vivarium for 3 days after arrival. On day 4, the procedure was effected. Animals were premedicated intramuscularly with atropine sulphate (0.04 mg/kg) and anesthesia was induced with intramuscular Telazol/Xylazine 4–6/2 mg/kg. The animals were placed in recumbence on their left side on a fluoroscopy table. Vital signs (heart rate, respiratory rate, and anesthetic depth) were continuously monitored during the procedure. Prophylactic antibiotic Enrofloxacin 2.5 mg/kg was administered intramuscularly before the procedure, after anesthesia.

**Figure 1:**
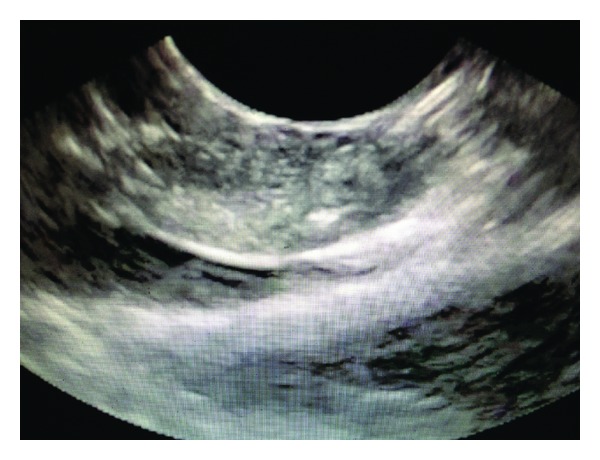
Endoscopic Ultrasound view of the EUS-RFA probe inserted into the porcine pancreas.

**Figure 2:**
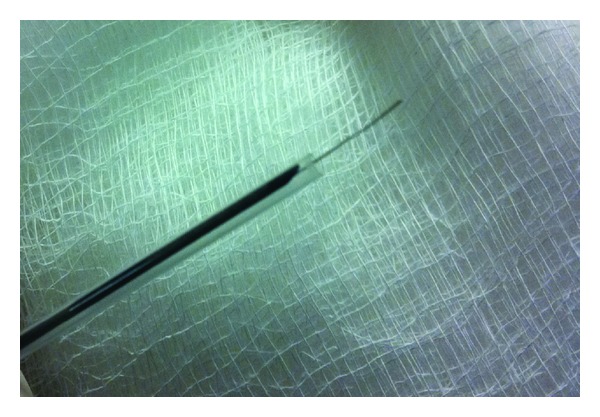
Habib EUS RFA probe.

The porcine pancreatic tissue was ablated with RFA after placing an EUS guided 19 gauge Wilson Cook needle into the pancreas in a transduodenal approach. The echoendoscope (Linear Endoscope (EG-3870UTK) 3.8 mm, Pentax Montvale, NJ, USA) was advanced through the mouth to the duodenal bulb and observed by ultrasonography of the pancreas. A 19-gauge needle (Wilson Cook, Winston-Salem, NC, USA) was inserted through the working channel of the endoscope into the pancreas (Figure 1). The needle was used to puncture the pancreas and the stylet was removed. The pilot RFA probe connected to RITA (Electrosurgical RF Generator) was then advanced through the needle into the pancreas. The pilot Habib EUS RFA probe (EMcision LTd., London, UK) is a 1 Fr wire (0.33 mm, 0.013′′) and has a working length of 190 cm (Figure 2). 

The RFA probe was applied with 6 mm of the probe exposed at 4 watts for 300 seconds (5 mins), 5 watts for 54 seconds (0.9 mins), and 6 watts for 12 seconds (0.2 mins). Then with 10 mm of the probe exposed in the pancreas, RFA was affected at 4 watts for 258 seconds (4.3 mins), 5 watts for 84 seconds (1.4 mins), and 6 watts for 48 seconds (0.8 mins). The wattage and exposure time was predetermined based on in vitro testing with a generator.

After procedure, yohimbe 0.3 mg/kg was given intravenously to hasten recovery from anesthesia and a fentanyl patch was given for analgesia. 

**Figure 3:**
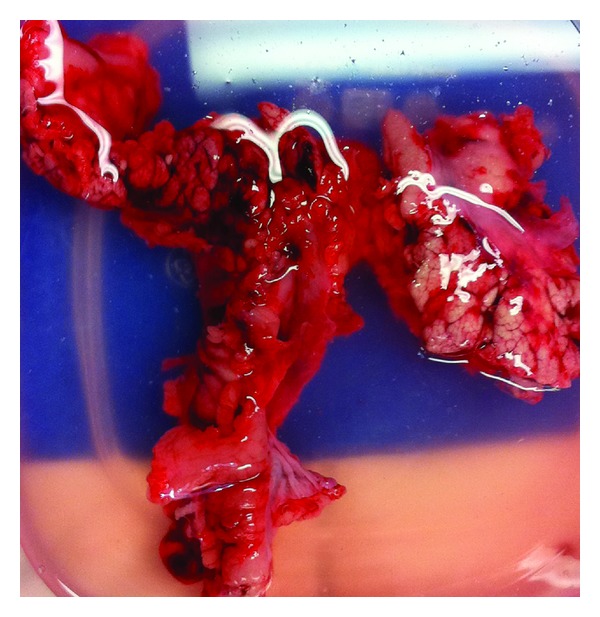
Excised porcine pancreas after euthanization.

Three days after procedure, blood was drawn to evaluate total bilirubin, alkaline phosphatase, cell blood count, and amylase. On the 6th day after procedure, the pigs were euthanized. The pancreas of the pigs were immediately excised surgically for gross examination of damage, tissue response, and histological analysis (Figure 3). 

## 3. Pathologic Examination

### 3.1. Histopathological Assessment

Pancreata were excised and fixed in neutral buffered formalin. The organs were serially sectioned at 3 mm intervals by a dedicated GI pathologist blinded to the procedure performed. Cross-sections were taken from the proximal pancreas (2-3 cm from the ampulla) and from the distal pancreas (2-3 cm from the tail end) by the pathologist. The sections were subjected to routine processing and paraffin embedding. Four-micron histologic sections were stained with hematoxylin and eosin (H&E). The presence of acute pancreatitis (cell necrosis) was assessed as an estimate of the percent area of acinar pancreatic tissue involved. 

## 4. Results

All 5 Yucatan pigs tolerated the RFA. The pigs did not display any abnormal behavior or signs of complications after procedure. 

Due to inadequate EUS visualization of the pancreas and repositioning difficulties with the probe in pig 1, the pancreas was ablated once for 5 minutes at 4 watts with 6 mm of the probe exposed. In pig 2, with 10 mm of the probe exposed, it was activated for 84 seconds (1.4 mins) at 5 watts; 48 seconds (0.8 mins) at 6 watts; twice for 258 seconds (4.3 mins) at 4 watts. The probe was activated 7 times instead of 6 times (10 mm, 4.3 minutes at 4 watts) in pig 2 to test for efficacy at a different site within the pancreas. 

### 4.1. Lab Results and Complications Assessment

Three days after procedure, blood was drawn to evaluate total bilirubin, alkaline phosphatase, cell blood count, and amylase. The values were within normal range, and the pigs did not display any symptoms or abnormal behavior. 

### 4.2. Tissue Analysis

**Figure 4:**
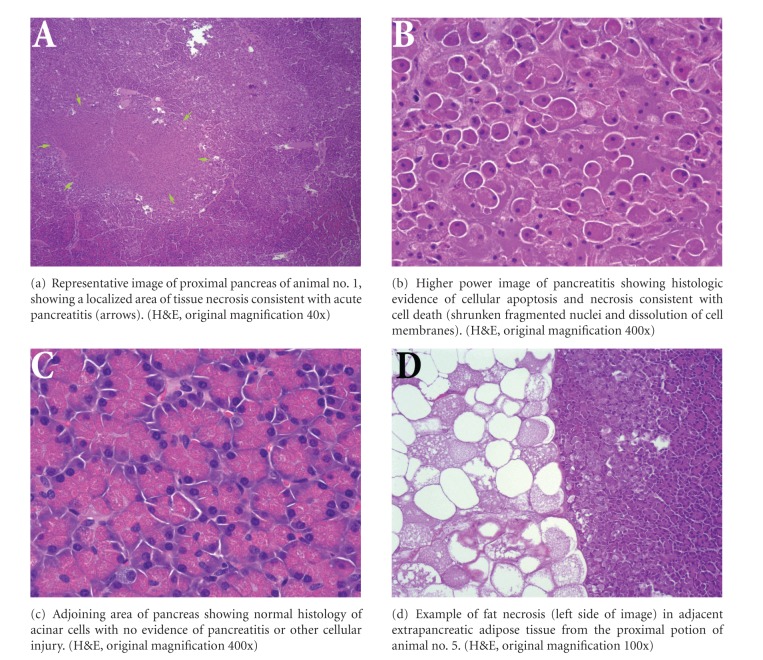
Pancreatic histology.

No gross abnormalities were noted during the serial sectioning of the pancreata. Examination of the representative histologic sections showed focal areas of acute pancreatitis as evidenced by necrotic change of acinar pancreatic tissue (Table 1 and Figure 4). Only one animal (no. 1) showed moderate levels of pancreatitis, with involvement of 20% of the proximal pancreatic tissue. The other animals showed much lower areas of tissue damage. In 3 of the 5 animals, the proximal pancreas showed greater levels of tissue injury than the distal pancreas, consistent with the proximity of the tissue to the procedure site. In one animal (no. 4), there was minimal (1%) pancreatitis in both the proximal and distal pancreas, and in one animal (no. 3), slightly more injury was seen in the distal pancreas versus the proximal pancreas (4% versus 1%). In all tissue sections examined, there was evidence of fat necrosis in intrapancreatic and/or extrapancreatic adipose tissue (Figure 4(d)). In pigs no. 3 and no. 5, fat necrosis around their pancreases was seen, indicating pancreatitis. However, the pigs had normal lab values and did not display any symptoms or abnormal behavior.

## 5. Discussion

Radiofrequency ablation (RFA) uses high-frequency alternating current to destroy solid tumors [9]. When attached to a generator, RF current is emitted from the exposed portion of the electrode and this current translates into ion agitation within the surrounding tissue, which is converted by friction into heat and induces cellular death by means of coagulation necrosis [12, 13]. Its minimally invasive approach and good tolerability are the advantages of using RFA [9].

RFA of the bile duct during endoscopic retrograde cholangiography (EndoHPB probe, London, UK) was used in 2 studies [8, 14] and seems to be efficacious and well tolerated. Also, percutaneous RF-induced tissue coagulation has been used in early clinical trials for the management of hepatocellular carcinoma [15] and hepatic [15, 16] and cerebral metastases [17]. 

EUS has been increasingly used for therapeutic purposes as it allows precise measurement of the location and size of the pancreatic masses and can be used to follow the area of ablation and help avoid surrounding structures. The potential advantage of ablation with EUS is the guidance by real-time imaging into a deeply located target such as the pancreas, which is extremely difficult to reach percutaneously [4]. EUS-RFA is a safe, effective, and well-recognized modality for the treatment of focal malignant diseases [18, 19]. 

In the studies of Wu et al., Van Goethem et al., and Lee et al., the bipolar probe was found to ablate with less collateral thermal damage than the monopolar system but with less efficiency overall [5, 20, 21]. A hybrid cryotherm probe (CTP) combines the bipolar RF ablation with cryotechnology [4] increasing RF-induced interstitial devitalization [22]. Carrara et al. [4] utilized the EUS-guided CTP in pigs and found the longer the application time, the greater the variation in lesion size; an application of 900 seconds induced a high complication rate in the healthy pancreas. The mean size of the ablation zone obtained in this experiment with the bipolar probe and a 300-second application was about twice as big as the ablation zone obtained with the monopolar system at 360 seconds [13]. The mortality was zero while the morbidity was significant with one (7%) symptomatic necrotic pancreatitis with peritonitis, one burn of the gastric wall, and four (28.5%) adhesions between the pancreas and the gut. The burn of the gastric wall was thought to be due to incomplete probe penetration of the gastric mucosa, which is thicker in pigs than humans.

Similar complications were seen in Goldberg et al. [13], where EUS-RF was applied for 6 minutes to normal pancreatic tissue of 13 Yucatan pigs with specifically modified 19-gauge needle electrodes (285 ± 120 mA) via a transgastric approach. One pig had mild hyperlipasemia, a focal zone of pancreatitis (<1 cm), and later a pancreatic fluid collection. Other complications included three gastric and one intestinal burn caused by improper electrode placement. In pigs killed immediately and 1 to 2 days after ablation, pathological examination showed discrete, well-demarcated spherical foci of coagulation necrosis measuring 8 to 12 mm in diameter surrounded by a 1 to 2 mm rim of hemorrhage. 

The complications seem to be associated with the duration of the ablation. The pancreas is very thermosensitive biological tissue and the thermal ablation of normal pancreas leads to an inflammatory response with edema and fibrotic and sometimes cystic transformation [4]. A major risk of massive necrosis seems to be related to multiple ablations that are in close proximity during the same treatment [5, 9].

The monopolar system was chosen in our EUS-guided ablation experiment over the bipolar due increased efficiency overall. This is remarkable considering that our ablation target was the head of the pancreas, where the sequelae of ductal trauma may be more significant [4]. Prior studies have shown that achieving maximum coagulation diameter in the liver, muscle, and intrahepatic tumor requires 6 minutes of RF application [23]. Thus, we aimed at concentrating around this duration time in our experiment. Currently achievable coagulation diameter is between 8 to 10 mm [4, 24–27] and so larger tumors might necessitate multiple needle insertions and RF applications [4]. Varadarajulu et al. in 2009 [28] achieved a complete coagulation necrosis of 2.6 cm diameter in the liver of 5 Yucatan pigs without damage to the surrounding parenchyma or vasculature utilizing EUS-RFA with a 19-gauge FNA needle fitted with an umbrella-shaped retractable needle electrode array. Noteworthy, this electrode array prototype with an umbrella diameter of 2 cm may be too large for the pancreas and even other organs [28].

In our study, there was evidence of fat necrosis in intrapancreatic and/or extrapancreatic adipose tissue. In two pigs (no. 3 and no. 5), fat necrosis around their pancreases was seen, indicating pancreatitis. However, in two of the pigs, EUS visualization was suboptimal; this may be due to a duodenal view of the pancreatic head not being very feasible in pigs because the stomach is longer than in humans and the pyloric muscle is very thick and difficult to pass [4]. In addition, a two-dimensional (2D) endosonography was used whereas a 3D ultrasound picture would improve the accuracy of the positioning of the probe [4] and potentially the visualization of the ablation site. Pancreatitis was achieved in both the proximal and distal pancreas even though our ablation target was the proximal pancreas. Therefore, some of the inflammatory changes of the pancreas could have been attributed to the needle insertion and not ablation alone. Pancreatitis was not detected between the proximal and distal sections of the porcine pancreas which may be related to the anatomy of the porcine pancreas. The area of necrosis could not be measured due to the limited necrosis induced. 

EUS-guided RFA appears to be well tolerated and most complications due to initial technical problems or differences between porcine and human anatomy [13]. RFA has been effective in the treatment of unresectable hepatic tumors and promising results have been obtained in tumors of the lung, bone, kidney, brain, breast, and prostate [9]. Thus, other applications of RFA exist but further studies in animals are needed to investigate the radiofrequency ablation of pancreatic tumor tissue with the monopolar probe prior to its use for palliation of unresectable malignant tumors of the pancreas. Additional modification of existent technology likely is needed to allow coagulation of greater volumes of tissue [13]. 

In conclusion, EUS-guided radiofrequency ablation of the pancreatic head with the monopolar probe through a 19-gauge needle was well tolerated in 5 Yucatan pigs and with minimum amount of pancreatitis. Contrary to the previous study conducted in porcine pancreas, our study has demonstrated that RFA can be delivered via EUS with minimal pancreatitis. Although the safety of EUS-RFA has been proven, the effectiveness of the monopolar probe in EUS-guided RFA in pancreatic cancer remains to be determined. Future refinements of the device with better visualization and higher energy should allow for greater ablation effectiveness without jeopardizing safety.

## Figures and Tables

**Table 1:** Scoring of histologic injury.

Animal #	Location	% acute pancreatitis	Fat necrosis
1	Proximal	20	Present
1	Distal	2	Present
2	Proximal	7	Present
2	Distal	0	Present
3	Proximal	1	Present
3	Distal	4	Present
4	Proximal	1	Present
4	Distal	1	Present
5	Proximal	5	Present
5	Distal	2	Present
